# Understanding the challenges of disaster victim identification: perspectives of Australian forensic practitioners

**DOI:** 10.1093/fsr/owad020

**Published:** 2023-06-23

**Authors:** Natasa Adamovic, Loene M Howes, Rob White, Roberta Julian

**Affiliations:** School of Social Sciences, College of Arts, Law and Education and Tasmanian Institute of Law Enforcement Studies (TILES), University of Tasmania, Hobart, Australia; School of Social Sciences, College of Arts, Law and Education and Tasmanian Institute of Law Enforcement Studies (TILES), University of Tasmania, Hobart, Australia; School of Social Sciences, College of Arts, Law and Education and Tasmanian Institute of Law Enforcement Studies (TILES), University of Tasmania, Hobart, Australia; School of Social Sciences, College of Arts, Law and Education and Tasmanian Institute of Law Enforcement Studies (TILES), University of Tasmania, Hobart, Australia

**Keywords:** disaster victim identification, disasters, forensic practitioners, postmortem phase, operational challenges

## Abstract

Disaster victim identification (DVI) is an important process in the aftermath of disasters to provide answers for the families and communities of victims. Australian forensic practitioners contribute to such processes internationally under difficult post-disaster circumstances. The aim of the study was to better understand the challenges experienced by forensic practitioners in international DVI operations. Participants (*N* = 20) included DNA analysts, fingerprint examiners, forensic odontologists, forensic pathologists, and mortuary technicians who had experience in DVI operations. Participants were interviewed about their experiences and perceptions of the challenges of DVI. The findings provide valuable insights into the types of DVI operations in which Australian forensic practitioners have been involved internationally. Thematic analysis of interview data resulted in five main themes: the post-disaster work environment; DVI management and processes; political and financial influences; teamwork in intercultural and interdisciplinary contexts; and confronting the emotional realities of DVI work. The analysis highlights the interrelated challenges associated with DVI operations in international contexts. Practitioners also provided suggestions for improvement, which generally aligned with the themes and reflected an ethos of learning and continuous improvement in DVI. Further research on education and training and capacity-development initiatives is warranted.

## Introduction

Disaster victim identification (DVI) is often regarded as a category of humanitarian forensic action [1]. Humanitarian forensic action refers to “the application of forensic science to humanitarian activities” [2]. Like the use of forensic science and forensic medicine in post-conflict environments, mass graves, or missing persons cases, their use in DVI is to restore identity to the deceased. The purpose of doing so is to provide dignity in death for victims and to provide answers to the family members and community about what has happened [2]. Geographical, geological, social, and political realities are a factor in the distribution of disaster incidents—and the associated DVI responses [3]. When a disaster is the result of terrorist or conflict-related activity (as opposed to a natural geological or climatic event), humanitarian forensic action can contribute to prosecutions to hold perpetrators to account [4]. The scale of disasters can vary from a small number of people involved in a remote helicopter crash to the widespread damage and mass fatalities associated with the 2004 Indian Ocean tsunami [5, 6].

The vital work of DVI in the aftermath of a disaster is a complex undertaking. Leading a disaster response is typically the responsibility of the government in the affected area [7, 8]. However, led by organisations such as the International Committee of the Red Cross and International Criminal Police Organization (INTERPOL) [2], the international community stands ready to assist in DVI, contributing highly specialised skills and multidisciplinary teams. Governments deploy teams when their nationals are among the dead as well as when requests for assistance are received from governments in affected areas. For large-scale incidents involving victims of multiple nationalities, responses involve people from multiple countries, agencies, and disciplines who must work towards a common goal under difficult circumstances. This raises difficult questions about the value of different human bodies in death as in life [3]. As Henry Quarantelli—a founding scholar of the social science of disasters—emphasised, disasters are inherently social phenomena and work in this field occurs within particular social contexts [9]. Similarly, researchers have recognised forensic science as a social phenomenon, arguing that a critical social sciences perspective provides important insights into differential impacts and outcomes of its use [10]. This perspective proposes that forensic science takes place within particular economic, political, and cultural contexts and there is human intervention at every stage. The application of forensic science is impacted by inherent power relations and contest over values and hierarchies of knowledge and expertise [10], in this case administrative and professional knowledge [3, 11]. In DVI, some of the inherent complexity is suggested by the humanitarian role and international context, as well as the post-disaster environment in which DVI occurs.

This paper reports the findings of a study that aimed to better understand the complexities of DVI. Specifically, it focuses on the challenges of DVI from the perspective of Australian forensic practitioners. The study adopted a critical forensic studies perspective, which is informed by social justice and human rights considerations in the use of forensic science to improve fairness for all [10]. Although the research did not focus on the experiences of victims’ families and communities *per se*, it aims to contribute to enhanced outcomes for such groups. Specifically, by identifying issues from practitioners’ perspectives, the findings from this study may stimulate further research to eliminate or alleviate issues and contribute to training and development to facilitate this important work. The paper begins with an overview of DVI processes, including some of the associated challenges. It then discusses the methodology of the study in which 20 Australian forensic practitioners from five forensic disciplines were interviewed about their experiences of DVI in international contexts. The paper concludes with a discussion of the findings and suggestions for further research that arise from this study.

## DVI processes

The process of DVI involves many different types of practitioners, such as photographers, radiologists, interview teams, property managers, investigators, liaison offers, logistics officers, and various forensic practitioners [8]. The forensic practitioners include forensic pathologists, forensic anthropologists, forensic odontologists, DNA analysts, and fingerprint examiners. Further forensic disciplinary specialists may be involved, depending on the nature of the incident. For example, after the 2002 Bali bombing, forensic chemists attended and established an innovative mobile laboratory to examine post-blast samples [12]. Deployment of forensic practitioners as part of a national team, where supervision and mentoring is provided, can offer valuable experience and the opportunity for vital skills transfer [13]. For example, after their first international DVI deployment (in response to the MH17 disaster), the Malaysian team reported valuable learning despite the many challenges [14].

To facilitate shared understandings of best practices in DVI, INTERPOL provides guidelines for the DVI process. The *INTERPOL Disaster Victim Identification Guide* [8] outlines the four phases of the DVI process, and the responsibilities associated with each one. Debriefs are sometimes considered a fifth phase [15], and although they are discussed by INTERPOL [8], they are not explicitly described as a phase.

### Phase 1—scene

Briefly, in Phase 1, the disaster scene should be treated as a crime scene. A scene management plan should be developed [8] and involve consultation with experts from the relevant forensic disciplines ([5, 8] see Part B Annexure 17, regarding forensic anthropology). Once the scene is safe to enter, crime scene and DVI specialists then commence their work, including the photographing, recording, and labelling of exhibits. Details are recorded using the INTERPOL Disaster Victim Recovery form. Coordination and communication are important aspects of the phase as human remains and property (e.g*.* clothing and jewellery) must then be transported and stored appropriately before they are examined at the mortuary [8].

In practice, although restricting access to a scene is desirable due to possible danger and the potential for recovery efforts to be complicated, the impacts of a disaster may be widespread and therefore it may not be possible to contain a scene [16]. For example, following the Bali bombing, well-intentioned volunteers did not always bag human remains separately, complicating later processes [17]. Similarly, after Malaysia Airlines Flight MH17 was downed in a conflict-affected region of Ukraine, the scene was not secure. The first responders in the aftermath of a disaster are often community members who have not been trained for the purpose. In recognition of this issue, a field manual has been developed to assist first responders and has been updated to incorporate more recent experience from the field [18].

### Phase 2—postmortem

Phase 2 is known as the postmortem phase and takes place at a mortuary. It may be possible to use an existing mortuary, or it may be necessary to establish a temporary one for the purposes of the DVI process [8]. For example, following the 2011 earthquake in Christchurch, New Zealand, the hospital that housed the mortuary was damaged. The national DVI team, members of the New Zealand Defence Force, and private contractors established a temporary mortuary in the hangar of a nearby military camp [19]. Refrigerated storage for bodies and human remains can be another pressing issue—particularly in hot and humid climates—to prevent rapid decomposition [16, 20, 21].

Once a mortuary is established, all human remains and personal effects are processed and examined by specialists from relevant disciplines. The postmortem INTERPOL DVI form is used to record details of unidentified human remains. Typically, one or more lines of several stations are established [22]. In the MH17 response, led by the Dutch team, human remains were transported to a temporary mortuary in Hilversum in the Netherlands, and five lines of five stations were established. The stations were personal items, fingerprints, DNA and autopsy, odontology, and quality control [6, 14]. Photography and descriptions began the process, then digital fingerprint capture technology was used [6]. The forensic pathologists and forensic anthropologists worked alongside DNA analysts at DNA stations, where all injuries were documented and appropriate DNA samples were taken (e.g*.* from bone). Then forensic odontologists made dental X-rays [6]. All human remains went to each station in the line, concluding with quality assurance, and were stored following analysis [14].

### Phase 3—antemortem

In Phase 3, the antemortem phase, missing persons’ data are collected [8]. In incidents where multi-national victims are involved, this effort is led by national teams in each affected country. It includes obtaining details of appearance and personal effects, often from family members, as well as collecting reference samples (e.g*.* of fingerprints and DNA) from their place of residence for comparison with postmortem data. It also involves locating medical and dental records in close coordination with relevant agencies. The antemortem (yellow) INTERPOL DVI form is used to record missing persons information.

The collection of antemortem data is facilitated by national record-keeping systems. In Thailand, following the Indian Ocean tsunami, delayed collection of antemortem data [16] and insufficient antemortem dental records (sometimes partly due to the devastation caused by the tsunami) were an issue [23]. Highlighting the disparities between countries, the Danish antemortem team had good access to dental records given that dental care was widely available to the population, and records were kept for 10 years [24]. Further, highlighting disparities within a country based on citizenship status, following the 11 September 2001 terrorist attacks on the World Trade Centre in the USA, it became apparent that an official list of the dead did not include all missing undocumented migrants, for a range of complex reasons [25]. The same was true of the Grenfell Tower fire in the UK in 2017, where migrant and asylum seeker communities were affected [26]. These factors impede communication with families and limit the collection of antemortem data, which can delay or deny identification.

### Phase 4—reconciliation

Finally, in Phase 4, the reconciliation phase, the postmortem and antemortem data (recorded on forms in previous phases) are compared to identify the victims [8]. Data entry, and databases (existing or specifically created) facilitate this process [6]. Specialised software was developed after the Bali bombing and before the Indian Ocean tsunami response [16]. Primary identification can be made from fingerprints, DNA, or odontology evidence. Additionally, an identification can sometimes be made from unique serial numbers of medical implants. Alternatively, secondary identification involves the use of personal description, medical findings, tattoos, and personal effects. When the identification board, headed by a coroner or equivalent within the country of the incident, is satisfied with identification data presented, a death certificate is prepared, and the remains can be repatriated to the country and family of the deceased [8].

It should be noted that visual identification from photographs or witnesses is notoriously unreliable—and is insufficient [8]. In fact, reliance on visual identification was used in the initial response to the tsunami in Thailand, but it led to some misidentifications—it was ultimately agreed that only primary identification (fingerprints, odontology, DNA) would be used [16]. Visual identification seemed possible after the 2011 earthquake and tsunami in the Tohoku region of Japan as cold weather slowed decomposition; however, it resulted in a number of misidentifications [27].

## The present study

As may already be apparent, much of the research on the challenges of DVI has focused on the responses to specific disasters. In a recent review of the field of disaster research, Wolbers et al. [28] found that there was a need for research to look beyond single cases to compare cases or to locate patterns across events or phases. Similarly, we note that much of the previous research on DVI has focused on reports on a national team’s experience in a multinational response, reflections on personal experience, or discipline-related challenges and forensic and technical innovations. These articles provide valuable insights and reflect a strong professional imperative for information sharing to advance the field. The present study responds to a call for research across disasters [28] and shares a focus on informing professional learning and development. It aimed to explore the challenges experienced by Australian practitioners from several forensic disciplines and across a range of international DVI operations. The purpose was to provide an overview of the challenges to contribute to understandings of the nature of this work and to identify aspects in need of further research.

## Methodology

### Sampling and participants

Ethical approval for the study was obtained from the University of Tasmania Human Research Ethics Committee [ID: H0024089]. Specific organisations in three jurisdictions were then contacted for permission to undertake research with members who had experience in international DVI operations. Organizational approvals were received from the Australian Federal Police for fingerprint examiners and DNA analysts, from the Tasmanian Department of Police, Fire and Emergency Management for DNA analysts based at Forensic Science Service Tasmania, and from Western Australia Police Force for fingerprint examiners.

Additionally, the National Institute of Forensic Science, which is a directorate of the Australian and New Zealand Policing Advisory Agency (ANZPAA) facilitated the research by requesting assistance from the ANZPAA DVI Committee (ADVIC). Its members include police representatives from each jurisdiction and scientific representatives from the biology Specialist Advisory Group, the odontology, anthropology, and mortuary Technical Advisory Groups, and the Royal College of Pathologists of Australasia. The chairs of forensic disciplines in ADVIC forwarded information about the study to their member forensic anthropologists, forensic pathologists, forensic odontologists, and mortuary technicians.

Once potential participants were nominated by their organisations, or had responded to the researcher directly, the first author contacted them *via* email, providing information and consent forms if they had not already received them. Mutually suitable times for interviews were arranged once consent forms were returned. A total of 20 participants were drawn from five forensic disciplines: DNA analysis (*n* = 4); fingerprint examination (*n* = 6); forensic odontology (*n* = 4); forensic pathology (*n* = 4); and mortuary services (*n* = 2). Of the 20 participants, 12 were female and eight were male forensic practitioners. Participants had experience in 1–11 separate DVI responses, with participation in 1 (*n* = 2), 2–4 (*n* = 9), 5–10 (*n* = 8), and 11 responses (*n* = 1). The most frequently mentioned DVI responses were the 2004 Indian Ocean tsunami response in Thailand (*n* = 15), the 2002 Bali bombing (*n* = 11), and the 2009 bushfires in Victoria (*n* = 8).

## Interview procedure

Most interviews were conducted *via* Zoom (*n* = 15), both to facilitate Australia-wide participation and to accommodate COVID-19 restrictions. The remainder of interviews (*n* = 5) were conducted face-to-face, in accordance with all recommended health measures. Participants were asked open-ended questions about their role and forensic expertise, their involvement and experience in DVI operations, the main challenges they had experienced during the DVI operation/s, and suggestions to improve DVI processes from their perspectives. Participants were asked follow-up questions as necessary to elicit further information or clarify points made and were encouraged to raise additional points that they considered relevant.

Interviews ranged in length from 41 min to 111 min (mean = 56 min). All interviews were recorded using an iPhone with the consent of participants. Interviews were transcribed verbatim. First, a list of DVI operations that participants had attended was compiled from the data. Next, interview transcripts were analysed thematically using the steps outlined by Braun and Clarke [29]. This involved the first author coding interview data, grouping similar codes to form proto-themes, and developing thematic maps [30], which were refined and clarified through discussion with the team. The analysis resulted in five themes and associated subthemes, as well as a summary of key suggestions for improvement of DVI processes from participants. In the following section, the illustrative quotes selected reflect the views of participants from across a range of forensic disciplines and DVI operations.

## Results

The results focus on three areas: an overview of DVI operations attended by participants, themes from interview data, and participants’ main suggestions for improvement.

### DVI experience

Interview data showed that collectively participants had experienced a broad range of DVI operations. As can be seen in Table 1, these included large-scale and well-known domestic and international DVI operations as well as smaller operations.

**Table 1:** Disaster victim identification (DVI) operations attended by participants.

Year(s) of event	Location	Type of disaster
1991–2001	Yugoslavia	War
1993	Texas, USA	Siege
1996	Tasmania, Australia	Mass shooting
1999	Kosovo	War
2002	Bali, Indonesia	Bombing
2003–2011	Iraq	War
2004–2005	Thailand	Tsunami
2005	Baghdad, Iraq	Air disaster
2005	Egypt	Bombings
2005	Comoros Islands	Air disaster
2005	Libya	Air disaster
2007	Myanmar	Cyclone
2007	Indonesia	Air disaster
2008	Philippines	Typhoon
2009	Victoria, Australia	Bushfires
2009	Samoa	Tsunami
2009	Papua New Guinea	Air disaster
2010	Congo	Air disaster
2010	Christmas Island	Identification of deceased asylum seekers
2011	Christchurch, New Zealand	Earthquake
2012	New Zealand	Balloon crash
2014	Ukraine	MH17 air disaster
2014–2016	West Africa	Ebola crisis
2015	Nepal	Earthquake
2019	New Zealand	Volcanic eruption
2019	Egypt	Bombings

### Themes developed from interview data

Drawing upon their experience, participants discussed various types of challenges in DVI operations. Themes and subthemes are summarized in Table 2.

**Table 2:** Themes and subthemes developed from thematic analysis of interview transcripts (*N* = 20).

No.	Theme	Subthemes	Number
1	The post-disaster work environment	Logistical challenges	18
		Lack of resources and equipment	15
		Feeling unprepared (often despite preparation)	15
2	DVI management and processes	DVI protocols	11
		INTERPOL forms	16
3	Political and financial influences	Political factors	12
		Financial resources	5
4	Teamwork in intercultural and interdisciplinary contexts	Relationships within and between teams	12
		Legal, cultural, and religious norms	16
		Language differences	8
5	Confronting the emotional realities of DVI	Reactions to conditions	13
		Encountering victims’ relatives	16
		Psychological and physical impacts	15

Number refers to the number of participants whose comments reflected the theme. DVI: disaster victim identification; INTERPOL: International Criminal Police Organization.

#### The post-disaster work environment

Reflecting experiences mentioned in previous literature [19, 22], participants explained that from the moment they were asked to assist in a DVI operation, they were confronted by immediate *logistical challenges*, such as getting to the disaster location and working in the post-disaster environment:

There’ll be logistical challenges you know that people haven’t got food, the power has gone down, the roads are flooded, the people haven’t got anywhere to live so they’re not focused on identifying the dead. It is very difficult to encompass the complexity of this when the numbers get large, and the disaster has affected the functioning of the society and the community. (Forensic Pathologist 1)

Also reflecting previous literature [16, 21], the DVI working environment was further complicated by a *lack of resources and equipment*:

In the Solomon Islands, we had to transport everything out to the remote sites by helicopter. Most of the places—there was no power at any of the places; there were no buildings, no running water. We had like creeks and the ocean and stuff, but no running water. And so, we had no decontamination facilities. I guess they were just all really remote, and everything pretty much had to be carried a fair distance by us—tables, bodies, bodies in bags, equipment, everything had to be carried to the site from the helicopter on our backs. So, we were quite limited in what we could take. And then we had to all be clean before we could get back on the helicopter, so it involved a swim in the ocean. (Fingerprint Examiner 8)

Participants reported *feeling unprepared (often despite preparation)* for the specific circumstances of the post-disaster environment, partly because each disaster was different and unexpected:

The Bali bombing … we were prepared, but we weren’t. There were so many things we could have and should have done better, and we learned. And then by the time we got to the bushfires, Victorian Bushfires, we used what we learnt in the Bali bombing with the Victorian bushfires. (Mortuary Technician 10)

Forensic practitioners therefore had to work out how to do their work differently, under extremely challenging circumstances, by building on experience.

#### DVI management and processes

The INTERPOL DVI Guide [8] aims to facilitate common processes internationally. However, reflecting previous studies [16, 17, 21], participants explained that *DVI protocols* were not always followed or well-understood by local authorities, at least in the initial stages of a response:

…No body bag is identified or has a number on it. So immediately, eight bodies from eight different locations—where the location might have said something crucial about who that dead body was, that’s immediately lost. A truck comes and the eight bodies get put on a truck. They go and pick up another eight bodies from another street—none of these are labelled and the bodies are taken off and put into a huge mass grave where lots of other bodies brought by lots of other trunks from lots of other locations are all mixed up in one big mass grave. And you have no idea at that point where any of these bodies have come from and at that point nobody has made a decision about [how to address the issue]. (Forensic Pathologist 1)

This sentiment was compounded by challenges in the lack of clarity in systems and processes in place:

When I got there, the handover was relatively short–very short really! It was just like, “Here’s the office, this is your team, this is the roster, these are the current files—do your best, we’ll see you later”. And then they went, so it wasn’t a lot, it didn’t take a whole day to handover or meet and greet people; you had to find your own ways. It was very rushed…it was like what I would imagine Wall Street would be like on a trading day—there are papers everywhere, noise, just chaos, really, in there. (Fingerprint Examiner 17)

This issue reflected an issue noted by Wright et al*.* [23] that when new members arrived, they would start searching for possible matches in cases that had been set aside by others because there was no mechanism for reporting confirmed exclusions. Additionally, the mandated *INTERPOL forms*, although necessary, were not always easy to use or responsive to the needs of the specific disaster situation:

. . . even the INTERPOL form needs to be tailored to the incident. And if you got an instance when people are massively burnt, e.g. everywhere is burnt, there’s no point in collecting certain information that the INTERPOL form would ask you, because that information isn’t going to be there. (Forensic Pathologist 2)

Despite the challenges of using the forms effectively, it was acknowledged that importantly, they helped to facilitate a common process.

#### Political and financial influences

Reflecting an understanding that many necessary negotiations take place at a higher (e.g*.* government) level [3], participants reported that many of the challenges faced were out of their own control. For example, the way that a DVI operation was managed was influenced by *political factors* and could require delicate navigation:

The politics depend on the jurisdictional power and the linkage between government and that particular lead agency. And then you’ve got what we can call powerful figures … So you have these very interesting, different political structures, which can completely change the way in which the DVI process is run [depending on] which one of them has sufficient political power to be in charge of the response. (Forensic Pathologist 2)

This kind of issue can be particularly relevant in developing countries [3, 16, 21]. Limited *financial resources* can increase reliance on international support in multiple domains, complicating questions of who is in charge (local authorities) and who is making resourcing decisions (foreign governments):

And in other (developing) countries, they certainly don’t have that sort of money to apply to DVI response. Therefore, we’ve got what we call scales of economy that are going to change the face of the DVI response, depending on which country you’re dealing with and their management style, the management culture, how much money is available, how much money or how much [in the way of] resources are provided to them by those that are assisting. (Fingerprint Examiner 19)

This issue reflects what occurred in Thailand, where the Australian Government funded the information centre [3]. Similarly, after a Norwegian company’s employees saw the conditions, they volunteered to build a temporary morgue. The Norwegian Government approved and the build received the blessing of local monks [16].

#### Teamwork in intercultural and interdisciplinary contexts

It was noted that DVI work “brings out the best and worst in people” (Forensic Odontologist 3); the best due to working hard to achieve a common goal, and the worst due to frustrations and difficulties being hard to bear. However, fostering good *relationships within and between teams* was important because, as noted in previous research [16], teamwork is essential in DVI not only within a particular national group, but also across disciplines and nationalities:

Well, it’s definitely teamwork. So, you really need to have a good team and they need to have the appropriate expertise underneath to be the type of personality that you can work with. We (also) need to have clear guidance on what’s going on. Because if you imagine going to a different country, it’s a different culture, might be different religions, different approaches to things, so we need parts of the teams going in who are going to be liaising with them and smoothing the way. (Forensic Odontologist 16)

Reflecting previous observations on differences in care for the dead [5], forensic practitioners noted that DVI operations could occur in countries or communities where unfamiliar *legal, cultural, and religious norms* could influence their work:

If you’re in New Zealand, speed is the essence of an investigation into a death because of the Maori sort of cultural context. And of course, that’s true for a number of religions as well—Judaism and Islam, and so on, both have issues around speed. And many of these are historical. If you actually think about it, they are more historical because of the issue around the body being preserved and so on. Whereas, for example, in Victoria and in elsewhere in Australia, a lot of the issues have been because they’ve transformed their death legislation—they’ve moved it to being more about consultation with the family. (Forensic Pathologist 2)

Additionally, whereas English was mostly used as a common language, differing levels of proficiency reportedly added to the complexities of communication (“and that has consequences” Forensic Pathologist 2). In reality, effective timely and accurate communication could pose challenges, with multiple *language differences*:

Because it was 20 or 30 nationalities. . . so the communication involved is intensive—multiple languages, communication channels, involving embassies and international police forces and different procedures and tracking people down. (Forensic Pathologist 1)

#### Confronting the emotional realities of DVI

Participants comments suggested that various different aspects of the DVI experience combined to make it overwhelming at times:

There is time away from home challenges. And time away from home compounded by seeing a never-ending stream of dead people or dead and decaying people and seeing bodies being opened up and jaws removed, then trying to fingerprint really rotten flesh and that constant stench of death. (Fingerprint Examiner 19)

This finding reflects research on disaster relief workers in general, which found that stress reactions can be generated, in part, by being away from home without one’s regular social support and experiencing culture shock amid the chaotic destruction [31]. Forensic practitioners reported that they were extremely motivated to do their very best to identify victims to help the affected families and communities; they were pleased to be able to assist in DVI. However, a central challenge reported was *encountering victims’ relatives*:

There were a lot of relatives there, quite a lot of people that were there looking for their loved ones, you know, they’re trying to find people, so it was quite daunting. You know, there’s a lot of people upset. (Forensic Pathologist 18)

This finding reflected sentiments from personal accounts [20] and from broader studies of disaster relief workers [32, 33]. As may be anticipated, some forensic practitioners reported *psychological and physical impacts* at the time of the DVI operation/s:

A few of the guys did say, you know, little things that would be happening to them like they felt mood changes or weren’t sleeping well, or felt very short tempered, and they thought it was just trying to assimilate this, but it was the first time they’d done one. (Forensic Odontologist 16)

Additionally, some reported that it was later that it may come up:

. . .I was a nervous wreck. Stomach was churning and I was on edge. I couldn’t work it out. What is this all about? … and I had a reaction that I didn’t know was sitting there except I had known when I came back, I couldn’t really talk about it. I got emotional like I still am a bit now, but it was really something. And that was probably close to 10 years later. So various stuff affects you—and you don’t realise. (DNA Analyst 7)

Overall, reflecting the broader body of research on the psychological and physical impacts on disaster relief workers [32, 34, 35], the experience of DVI had the potential to expose practitioners to various unsettling and potentially traumatising experiences.

### Participants’ suggestions for improvement

Participants (*n* = 18) provided suggestions for how the DVI process could be improved in general. They focused on several key areas as follows:

(i) Planning effectively for disasters (e.g*.* knowing in advance where to acquire generators and refrigerated containers to store human remains; preparing for instances of limited power sources);
(ii) Developing protocols (e.g*.* ensuring that norms are established in advance in terms of how various governments, NGOs, and agencies can best work together; further standardising DVI practices internationally; finding ways to increase the availability of, and access to, antemortem data);
(iii) Continuous learning and improvement (e.g*.* capturing the insights of leaders and those with first-hand knowledge to shape future practices; undertaking related research on identification strategies under different post-disaster circumstances; innovating with the use of technology and creative solutions); and
(iv) Managing expectations (e.g*.* communicating with those involved in the recovery phase about which samples to collect, and communicating with authorities about issues with resources or obstacles that may reduce the effectiveness of the work).

Overall, participants’ suggestions align with the themes discussed in the previous section. They reflect initiatives to learn and improve, share knowledge, and refine protocols after further DVI experience [18].

## Discussion

DVI work clearly takes place in complex social and political circumstances. The aim of this study was to obtain insights into the challenges faced by Australian forensic practitioners in DVI across forensic disciplines and disaster incidents. Due to the participants’ disciplinary expertise and roles, the study focused mainly on the postmortem phase of the DVI process. Although the list of DVI incidents generated from interview data represents only those attended and mentioned by the participants in this study, it gives some insight into the scope of Australian forensic practitioners’ involvement in DVI within and beyond the region. Additionally, the study identified five interrelated themes that provide a window into the challenges faced internationally by forensic practitioners in DVI. The findings may be helpful in making practitioners aware of some of the challenges others have faced in DVI, and for generating awareness more broadly among policymakers, members of organisations involved in disaster responses, and the broader public. The themes highlight various challenges that were interrelated in complex ways.

The first theme of *the post-disaster work environment* highlighted challenges of logistical issues, a lack of resources and equipment, and a sense of lacking preparation for what would be encountered—that meant that it was not possible for DVI work to commence immediately in the way that it otherwise might. This was perhaps the most prominent theme for participants in this study. These concerns resonate with the findings of previous studies [16, 19, 21, 22]. The second theme of *DVI management and processes* reflects participants’ sentiments on the necessity of good management to facilitate a smooth process. The present study also identified issues in the handover between rotations. These findings reflect a body of literature on the need for agreed ways of working in the aftermath of disasters [16, 18]. These kinds of occupational stressors have been found to affect the psychological wellbeing of disaster relief workers [36]. The development of INTERPOL guidelines, first responder guidelines, and regular updates of such documents are important to facilitate shared processes. However, it is also important to consider which ways are agreed and who benefits *versus* who is left behind in such agreements [3, 11].

Some of the reasons that it can be challenging to implement smooth processes are understood with reference to the third and fourth themes. The theme of *political and financial influences* reinforces that disaster-related issues are coupled with pre-existing disparities in countries’ wealth and governments’ access to resources. The theme reflects discussion by authors such as Scanlon [16] and Merli and Buck [3]. Not only is humanitarian forensic action a necessary action to support victims of a disaster, but also there is a need for capacity development among new practitioners [13] and to share knowledge within and between countries [1] so that DVI can be managed with requisite skills and strategies. The fourth theme of *teamwork in intercultural and interdisciplinary contexts* recognises that participants often undertake DVI work in unfamiliar terrain in terms of language, culture, and legal requirements. It reflects previous literature that suggests the need for briefings on the different traditions in care for the dead [5]. Helpfully, some authors have aimed to explain religious and cultural practices in caring for the dead, some of which may clash with scientific approaches to DVI [37–40], noting that premature burial or cremation after a disaster may also be motivated by a fear of public health consequences [41]. These themes add a layered explanation to the challenges of finding ways of working together under difficult circumstances.

The fifth theme of *confronting the emotional realities of DVI* reflects practitioners’ sentiments about the most difficult aspects of DVI from their personal perspectives. The pain for families of ambiguous loss, where the fate of a missing person is unknown, has been well documented in research literature [42, 43]. The present findings suggested that for some forensic practitioners, a heightened awareness of that pain contributed to perceived pressure and added grief, reflecting previous research [20, 32]. The theme also reflected the recognition that DVI work could expose practitioners to psychological (and physical) impacts. This finding highlights the importance of studies that suggest ways for organisations to assist practitioners to maintain psychological and physical health, identify protective factors, or improve treatment opportunities for practitioners (e.g*.* see reviews by [34–36]). The practitioners in this study expressed a strong commitment to this work, and their positive feelings about participating in it resonate with previous findings [32, 34]. Finally, *practitioners’ suggestions for improvement* reinforced practitioners’ recognition of the value of continuous improvement and ongoing learning, as well as the need for research and innovation in DVI.

## Conclusion

This cross-sectional study aimed to contribute to an understanding of the challenges faced by Australian forensic practitioners in DVI operations across a range of disasters. By identifying challenges for practitioners, the aim was to indirectly facilitate enhanced support for victims’ families and communities. Based on interviews with 20 practitioners with DVI experience from five forensic disciplines, the study revealed the involvement of Australian forensic practitioners in a range of DVI operations. The themes highlight the complexities of the DVI process, and the overlapping nature of challenges faced by forensic practitioners. The most prominent challenges include working together effectively in complex and often chaotic post-disaster environments, despite differences in political and financial, and cultural and legal norms. The findings suggest that forensic practitioners value ongoing efforts to improve and develop processes. Research on training, development, and capacity-building initiatives, both nationally and internationally, would be valuable to ensure that forensic science can be used as equitably as possible to assist families and communities in the aftermath of disasters.
